# Risks of Cardiac Arrhythmia Associated with COVID-19 Vaccination: A Systematic Review and Meta-Analysis

**DOI:** 10.3390/vaccines11010112

**Published:** 2023-01-03

**Authors:** Mohammed H. Abutaleb, Hafiz A. Makeen, Abdulkarim M. Meraya, Saad S. Alqahtani, Bayan Ibrahim Al-Mass, Reaam Omar Aljazaeri, Bushra Dhuhayyan Alhazmi, Afnan Mohammed Noor Kalakattawi, Ahmed Ali Alajam

**Affiliations:** 1 Pharmaceutical Care Department, King Fahad Central Hospital, Jazan Health Affairs, Ministry of Health, Jazan 82912, Saudi Arabia; 2 Pharmacy Practice Research Unit, Department of Pharmacy Practice, College of Pharmacy, Jazan University, Jazan 82912, Saudi Arabia; 3 College of Clinical Pharmacy, King Faisal University, Alahsa 31982, Saudi Arabia; 4 College of Pharmacy, Aljouf University, Sakaka 72311, Saudi Arabia; 5 College of Clinical Pharmacy, Northern Border University, Arar 91431, Saudi Arabia; 6 College of Clinical Pharmacy, Taif University, Taif 21974, Saudi Arabia; 7 Pharmaceutical Care Department, Aldarb General Hospital, Jazan 89876, Saudi Arabia

**Keywords:** COVID-19, vaccine, arrhythmia, incidence, meta-analysis

## Abstract

This systematic review and meta-analysis aimed to summarize the current evidence regarding the association between coronavirus disease 2019 (COVID-19) vaccination and the risk of cardiac arrhythmia. MEDLINE, via PubMed and OVID, Scopus, CENTRAL, and Web of Science were searched using the relevant keywords to identify the relevant citations. Comprehensive Meta-analysis and Review Manager 5.4.1 were used for all the statistical analyses. Seventeen studies (n = 567,033,087 patients) were included. The pooled analysis showed that the incidence of cardiac arrhythmia post-COVID-19 vaccination with Pfizer, Moderna, AstraZeneca, CoronaVac, and Sinopharm was 0.22%, 95% CI: (0.07% to 0.66%), 0.76%, 95% CI: (0.04% to 12.08%), 0.04%, 95% CI: (0.00% to 0.98%), 0.01%, 95% CI: (0.00% to 0.03%), and 0.03%, 95% CI: (0.00% to 18.48%), respectively. Compared to CoronaVac, Pfizer, Moderna, AstraZeneca, and Sinopharm had a higher incidence ratio rate (IRR; 22-times, 76-times, 4-times, and 3-times higher), respectively. Likewise, Pfizer, Moderna, and AstraZeneca showed a higher IRR than Sinopharm (7.3-times, 25.3-times, and 1.3-times higher). The current evidence shows that the incidence rate (IR) of cardiac arrhythmia post-COVID-19 vaccination is rare and ranges between 1 and 76 per 10,000. mRNA vaccines were associated with a higher IR of arrhythmia compared to vector-based vaccines. Inactivated vaccines showed the lowest IR of arrhythmia.

## 1. Introduction

Different treatment options have been investigated since the inception of the coronavirus disease 2019 (COVID-19) pandemic in late 2019, including immunotherapy, nucleoside analogs, and others [1,2]. To prevent severe acute respiratory syndrome coronavirus 2 (SARS-CoV-2) infection or to lessen the severity of the disease, with the ultimate aim of reducing mortality and morbidity, researchers have investigated a variety of platforms for vaccine development, including protein-based and viral-based vaccines [3,4].

There were 128 vaccines under clinical development as of 22 October 2021, with another 194 in the pre-clinical stage [5]. B-cell response, helper T cells, and cytotoxic cells are prompted by COVID-19 mRNA vaccines such as BNT1626b2 (i.e., Pfizer/BioNTech vaccine) and mRNA1273 (i.e., Moderna vaccine) [6]. Regarding vector vaccination, the COVID-19 virus’s genetic material is inserted into a modified form of another virus, which facilitates the delivery of genetic material from the COVID-19 virus that induces copies of the S protein [7]. The production of antibodies and protective white blood cells is triggered once S proteins are presented on the surface of the body cells. The Johnson & Johnson (Ad26.COV2.S) and Oxford and AstraZeneca (ChAdOx1) COVID-19 vaccines are vectors [8]. Protein subunit vaccines use fragments of protein from the disease-causing virus to trigger protective immunity against it. Studies have revealed that these protein vaccines include S proteins that are not pathogenic. Once the S proteins are recognized by the immune system, antibodies and protective white blood cells are generated [9]. The Novavax COVID-19 vaccine (NVX-CoV2373) is one of these protein-based vaccines. Since the approval of these vaccines, regardless of their type, several reports worldwide have emerged, documenting various adverse events, including local and systemic reactions.

Myalgia, headache, fever, edema, erythema, and pain at the injection site were the most common local or systemic reactions observed [10,11,12]. However, a number of serious and potentially fatal adverse events and complications have been documented in association with COVID-19 vaccinations, including venous thromboembolism, stroke, pulmonary embolism, cardiac arrhythmias, and myocardial infarction [13,14,15]. The unfavorable cardiac outcomes linked to COVID-19 vaccinations can range from inflammations (such as pericarditis or myocarditis) to thrombosis and ischemia [15,16]. Several systematic reviews and meta-analyses have investigated the risk of myocarditis/pericarditis, thrombocytopenia, and thrombosis in vaccinated COVID-19 individuals [17,18,19,20,21]. However, to the best of our knowledge, there is no large-scale study or meta-analysis that investigates the association between COVID-19 vaccination and cardiac arrhythmia. Therefore, in this systematic review and meta-analysis, we aimed to summarize the current evidence regarding the association between COVID-19 vaccination and the risk of arrhythmia.

## 2. Methods

The Preferred Reporting Items for Systematic Reviews and Meta-Analyses (PRISMA) checklist and Cochrane handbook for systematic reviews of interventions were followed in the reporting of this study [22,23]. The study protocol (CRD42022371085) was registered on PROSPERO.

### 2.1. Eligibility Criteria

The studies were included based on the following criteria:

Population: studies that were conducted on the individuals who received any type or dose of the COVID-19 vaccine.

Intervention and comparison: Pfizer-BioNTech, AstraZeneca, CoronaVac, Janssen, Sinopharm, or Moderna vaccines.

Outcomes: studies reported cardiac arrhythmia as an adverse event of one of the aforementioned vaccines. 

Studies design: observational studies (case–control, cohort, and cross-sectional).

We excluded case reports, conference abstracts, and non-English studies.

### 2.2. Information Sources and Search Strategy

On 22 September 2022, we searched the following databases: MEDLINE via PubMed and OVID, Scopus, CENTRAL, and Web of Science, using the relevant keywords to identify the relevant citations. The MeSH keywords used were “Cardiac Arrhythmias, COVID-19, COVID-19 Vaccines, 2019-nCoV Vaccine mRNA-1273, ChAdOx1 nCoV-19, BIBP COVID-19 vaccine, BNT162 Vaccine, Ad26COVS1, sinovac COVID-19 vaccine, CoronaVac vaccine, and BIBP COVID-19 vaccine”. These databases were searched from inception to the date of search. Moreover, the reference lists of all the included citations were searched. The retrieved citations were imported to EndNote X9 software, and duplications were removed.

### 2.3. Selection Process

Using Microsoft Excel software, a screening sheet was created. Study ID, publication year, title, abstract, keywords, DOI, and URL were all included. The selection process was undertaken using a two-step screening technique by three independent reviewers (B.I.A, R.O.A, and B.D.A). Step one included screening the title and abstract of all the studies found via the literature search to determine which studies might proceed to step two (full-text screening), where reviewers would read and assess whether each study met the eligibility criteria. Any disagreement between the reviewers was resolved by the judgment of the study supervisor (M.A). 

### 2.4. Data Items and Collection Process

Four independent reviewers extracted the following data from the included studies to an offline pre-prepared Excel sheet: demographic data of the included patients (age, gender, and residency), study characteristics (study groups, study duration, total sample size, country, and main findings) and outcomes (incidence rate of arrhythmia following COVID-19 vaccination). 

### 2.5. Risk of Bias and Quality Assessment 

Using the National Institutes of Health (NIH) quality assessment tool for observational cohort, case–control, and cross-sectional studies, two authors (A.M.N and A.A.A) independently evaluated the risk of bias and the quality of each included article. Reviewers can critically evaluate the internal validity of research using this tool. Studies were deemed to be “good,” “fair,” or “poor”. When the authors disagreed on a rating, a third author (M.A) resolved any disagreements. 

### 2.6. Data Synthesis

The incidence of adverse events was calculated using the random-effects model with a 95% CI. Using the I^2^ statistic, we calculated the percentage of heterogeneity and inconsistency between studies, with values of 25%, 50%, and 75% deemed as low, moderate, and high, respectively. The random-effect model was employed if the heterogeneity was considerable and if I^2^ > 50%; otherwise, the fixed-effect model was utilized. Comprehensive Meta-analysis software was used for all the statistical analyses (Biostat, Inc., CMA; USA: version 3.3.070, Florida, 33618, United States). To assess the difference between Pfizer-BioNTech and AstraZeneca in terms of AEs, we used the Review Manager 5.4 software to calculate the risk ratio (RR) between both groups using the Mantel–Hansel model. 

## 3. Results

### 3.1. Study Selection

**Figure 1:**
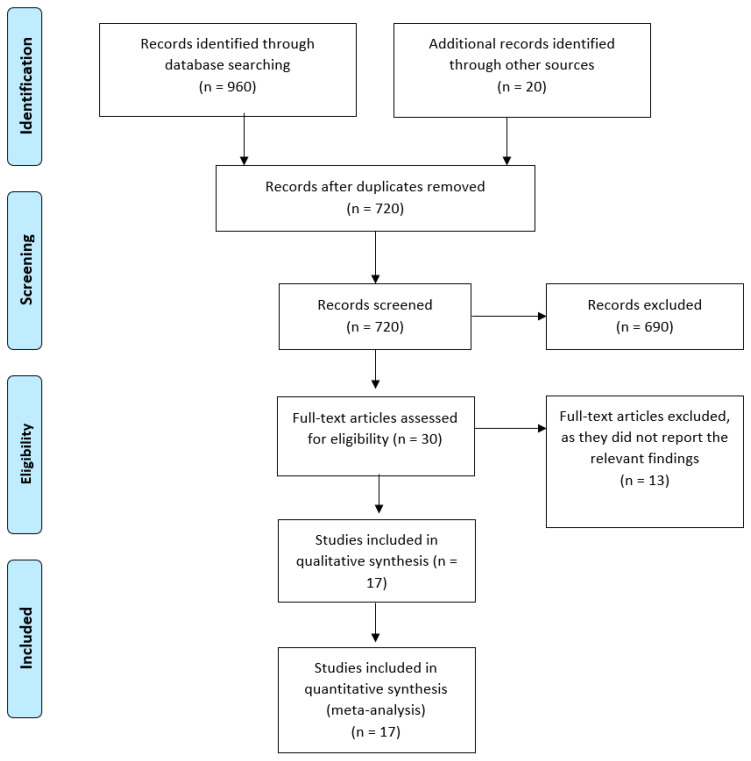
PRISMA flow diagram.

Based on our literature search, we found a total of 980 relevant citations. After removing the duplications, 720 articles underwent title/abstract screening. Then, 690 studies were deemed to be ineligible using our criteria. Full-text screening was performed on 30 articles, and only 17 studies (n = 567,033,087 patients) were included in the qualitative (systematic review) and quantitative synthesis (meta-analysis) [24,25,26,27,28,29,30,31,32,33,34,35,36,37,38,39,40]. Figure 1 shows the PRISMA flow diagram of the included studies.

### 3.2. Characteristics of Included Studies and Patients

Three studies were cohort studies, three were cross-sectional, eight were retrospective studies, two were controlled case-series studies, and one was a subgroup analysis study. Four studies were multicentric studies, three were from the USA, three were from Israel, one was from Malaysia, one was from Saudi Arabia, one was from Singapore, one was from Turkey, one was from India, one was from China, and one was from England. The Pfizer-BioNTech vaccine was reported in 12 studies, AstraZeneca in 3, CoronaVac in 3, and Moderna in 7. The age of the included patients ranged between 12 and 90 years old. Both males and females were represented in the included studies. Table 1 summarizes the baseline characteristics of the included studies and patients. 

### 3.3. Quality of the Included Studies

Based on the NIH quality assessment tool for observational studies, about 88.2% of the studies were deemed as “Good,” and 11.8% were deemed as “Fair”.

### 3.4. Meta-Analysis

#### 3.4.1. Incidence of Cardiac Arrhythmia

**Figure 2:**
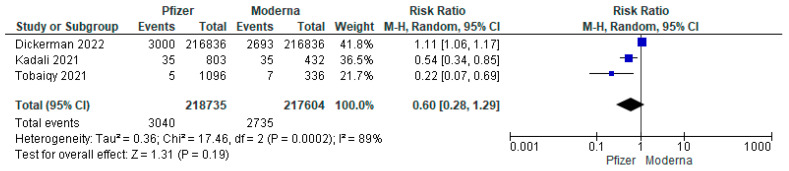
The RR of arrhythmia post-Pfizer COVID-19 vaccine compared to Moderna.

The pooled analysis of 12 studies showed that the incidence of cardiac arrhythmia post-Pfizer COVID-19 vaccine was 0.22%, 95% CI: (0.07% to 0.66%), which means that the incidence rate was 22 per 10,000 persons (Table 2). The random-effect estimate of four studies demonstrated that the pooled incidence of arrhythmia post-Moderna COVID-19 vaccine was 0.76%, 95% CI: (0.04% to 12.08%). Compared to Pfizer, the Moderna vaccine had an incidence rate ratio (IRR) of 3.45, meaning that the group that received the Moderna vaccine had a 3.45-times higher risk of developing cardiac arrhythmia than Pfizer. This finding was supported by the lower RR of cardiac arrhythmia associated with the Pfizer vaccine compared to the Moderna vaccine; however, the difference was not statistically significant (RR = 0.60, 95% CI: 0.28 to 1.29; Figure 2). 

**Figure 3:**
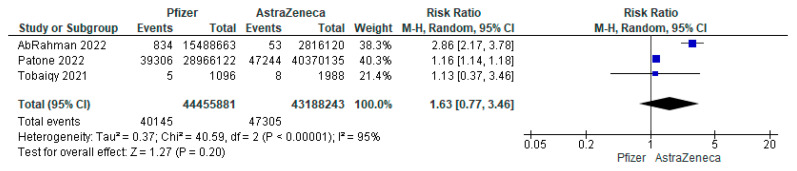
The RR of arrhythmia post-Pfizer COVID-19 vaccine compared to AstraZeneca.

**Figure 4:**
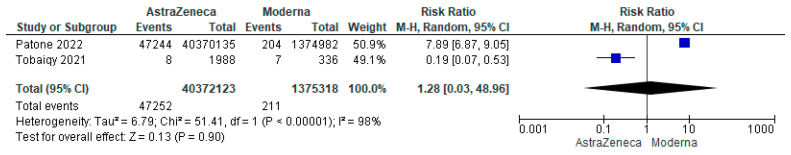
The RR of arrhythmia post-AstraZeneca COVID-19 vaccine compared to Moderna.

The pooled analysis of three studies showed that the incidence of arrhythmia post-AstraZeneca vaccine was 0.04%, 95% CI: (0.00% to 0.98%). The IRR arrhythmia post-Pfizer or Moderna COVID-19 vaccines was 5.5-times and 19-times higher than the IRR of arrhythmia post-AstraZeneca vaccine. Similarly, the pooled RR showed that the Pfizer vaccine had a non-significantly higher RR compared to AstraZeneca (RR = 1.63, 95% CI: 0.77 to 3.46; Figure 3). On the other hand, AstraZeneca and Moderna had a comparable RR (1.28, 95% CI: 0.03 to 48.96; Figure 4).

**Figure 5:**
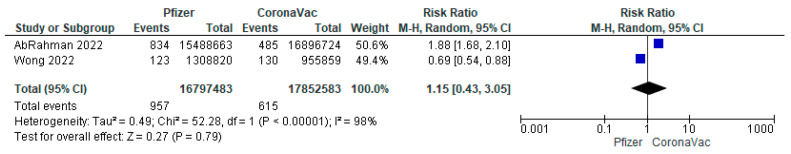
The RR of arrhythmia post-Pfizer COVID-19 vaccine compared to CoronaVac.

The random-effect model showed that the incidence of arrhythmia post-CoronaVac and Sinopharm vaccines was 0.01%, 95% CI: (0.00% to 0.03%) and 0.03%, 95% CI: (0.00% to 18.48%), respectively. Compared to CoronaVac, Pfizer, Moderna, AstraZeneca, and Sinopharm had a higher IRR (22-times, 76-times, 4-times, and 3-times higher), respectively. Likewise, Pfizer, Moderna, and AstraZeneca showed a higher IRR than Sinopharm (7.3-times, 25.3-times, and 1.3-times higher). The head-to-head comparison showed that there was no significant difference between the Pfizer and CoronaVac vaccines in terms of RR of arrhythmia (RR = 1.15, 95% CI: 0.43 to 3.05; Figure 5).

#### 3.4.2. Subgroup Analysis

The subgroup analysis according to the study design, sample size, and study setting is presented in Table 3. People from North America had the highest incidence of cardiac arrhythmia post-Pfizer COVID-19 vaccination at 11.95%, 95% CI: (4.92% to 18.98%), followed by Europe at 0.136%, 95% CI: (0.134% to 0.137%), the Middle East at 0.022%, 95% CI: (0.001% to 0.046%), and Asia at 0.006%, 95% CI: (0% to 0.009%). Studies that included a sample size larger than 2000 individuals reported a much lower incidence of cardiac arrhythmia post-Pfizer vaccine than those who included less than 2000. Regarding the study design, cross-sectional studies reported the highest incidence of cardiac arrhythmias compared to cohort studies, case-series studies, and retrospective studies.

## 4. Discussion

The current systematic review and meta-analysis showed that the IR of cardiac arrhythmia post-COVID-19 vaccination is rare and ranges between 1 and 76 per 10,000. mRNA vaccines, including Moderna and Pfizer, were associated with a higher IR of arrhythmia than vector-based vaccines. On the other hand, inactivated vaccines, such as CoronaVac and Sinopharm, showed the lowest IR of arrhythmia. Due to the absence of head-to-head comparison in the majority of the included studies and the non-significant difference in the RR between the studied vaccines, further studies are required to validate these findings. We did not find enough data about the risk of arrhythmia associated with the Johnson & Johnson vaccine to be pooled in one meta-analysis. However, the results of individual studies demonstrated that arrhythmia was one of the largest relative risks associated with COVID-19 vaccines, besides thromboembolic events, sexual organ reactions, ocular, gastrointestinal, constitutional, hemorrhage, coagulation, and other cardiovascular events. However, arrhythmia was not reported among the ten most frequent cardiovascular events attributed to the Moderna, Pfizer, or Johnson & Johnson vaccines. On the other hand, they showed that the Johnson & Johnson vaccine was associated with the lowest RR of arrhythmia when compared with Moderna and Pfizer (RR = 17.77, 95% CI: 15.48 to 20.39) vs. (RR = 56.87, 95% CI: 49.77 to 64.99 and RR= 70.80, 95% CI: 61.98 to 80.88), respectively [24]. Our findings were limited by the significant heterogeneity between the included studies. There were several causes for this heterogeneity, including the different population characteristics, study setting, study design, and administered vaccine doses. We found that North American studies had the highest incidence of arrhythmia post-Pfizer vaccine, compared to the Asian or Middle Eastern studies. This finding was consistent with the global reports that showed that the American population is more susceptible to cardiac arrhythmia compared to the European and Asian populations [41]. Moreover, we found that the self-reported incidences of cardiac arrhythmia were much higher than those based on clinician diagnosis.

Regarding the comparison between the Pfizer COVID-19 vaccine and other vaccines, AbRahman et al. conducted a controlled case-series study, comparing CoronaVac, Pfizer, and AstraZeneca. Their findings showed that among the patients who developed new-onset arrhythmia, 60.7% received the Pfizer COVID-19 vaccine, 35.3% received CoronaVac, 3.9% received AstraZeneca, and 0.2% received other vaccines. The event rate of arrhythmia per 1 million vaccinated persons was 95.47, 51.76, and 26.92, following Pfizer, CoronaVac, and AstraZeneca vaccines, respectively. However, the IRR results showed that those who received CoronaVac had the lowest IRR of arrhythmia compared to Pfizer and AstraZeneca (1.15, 95% CI: (0.96 to 1.17) vs. 1.16, 95% CI: (1.07 to 1.26) and 1.36, 95% CI: (0.95 to 1.94), respectively). Most of these patients were over 60 years old (66.5%). About 55.1% of the patients received the first dose of the vaccine, and 44.9% experienced arrhythmia after the second dose. Almost half of the patients already had hypertension, 16% had diabetes, and 12% had other heart diseases. They concluded that they could not highlight a significant association between cardiac arrhythmia and COVID-19 vaccines; however, arrhythmia is an AESI of concern [39]. According to Wong et al., there was no significant difference between CoronaVac and Pfizer in terms of the IRR of arrhythmia, CAD, or MI [37]. Compared to Moderna, the Pfizer vaccine induced a higher risk of arrhythmia (7.5 per 10,000 persons, 95% CI: 1.9 to 11.5 events) [27]. 

Although the numbers may not be indicative of the true situation, given that the Pfizer-BioNTech COVID-19 vaccine was the first to receive emergency approval from the US-FDA, and thus a large number of doses have been administered up until the period of analysis, one possible explanation is a shorter interval between the two doses, as shown by Kaur et al. [32]. The Case Series Drug Analysis Print published by Pfizer-BioNTech for their COVID-19 vaccine as of May 28, 2021, reported a total of 2342 cardiac AEs, among which 1098 events were classified as palpitations, 16 events were classified as acute myocardial infarction, 38 events as arrhythmia, 24 events as cardiac failure, 46 events as angina pectoris, 32 events as sinus tachycardia, 63 events as cardiac flutter, 62 events as cardiac arrest, 108 events as atrial fibrillation, and 466 events as tachycardia [42]. The rhythm disorders, as reported by AstraZeneca for their COVID-19 vaccine, included 1763 events of palpitations, 6 events of unstable angina, 8 events of supraventricular tachycardia, 8 events of tachyarrhythmia, 21 events of extrasystoles, 34 events of sinus tachycardia, 43 events of arrhythmia, 78 events of atrial fibrillation, and 622 events of tachycardia [43].

In terms of ECG changes associated with COVID-19 vaccines, Truong et al. enrolled 139 adolescents and young adults with 140 episodes of suspected myocarditis who received Pfizer, Moderna, and Janssen vaccines. Their findings showed that 97 patients presented with ECG changes and arrhythmias, including ST- or T-wave changes/elevation (97.9%), complete heart block (0.7%), first-degree atrioventricular block (0.7%), premature atrial contractions (0.7%), atrial tachycardia (0.7%), low-voltage QRS (3.6%), and non-sustained ventricular tachycardia (5%) [35]. 

When compared with non-vaccinated patients, the Pfizer vaccine was associated with non-statistically significant lower RR of arrhythmia (RR = 0.89, 95% CI: 0.74 to 1.04), according to Barda et al. They also demonstrated that vaccination against SARA-CoV-2 was associated with substantially lower RR of arrhythmia compared to infected patients, who showed a significantly high RR of arrhythmia (RR = 3.83, 95% CI: 3.07 to 4.95; 166.1 events per 100,000 persons) [40].

This is the first systematic review and meta-analysis that investigate the incidence rate of arrhythmia following COVID-19 vaccines. However, we acknowledge that our study has some limitations, including the small number of included studies, the significant heterogeneity, the inability to conduct a subgroup analysis for all vaccines, and the lack of head-to-head comparison in the majority of the included studies. 

In conclusion, the current evidence shows that the IR of cardiac arrhythmia post-COVID-19 vaccination is rare and ranges between 1 and 76 per 10,000. mRNA vaccines, including Moderna and Pfizer, were associated with a higher IR of arrhythmia compared to vector-based vaccines. On the other hand, inactivated vaccines, such as CoronaVac and Sinopharm, showed the lowest IR of arrhythmia. Due to the absence of head-to-head comparison in the majority of the included studies and the non-significant difference in the RR between the studied vaccines, further studies are required to validate these findings. 

## Figures and Tables

**Table 1:** Summary of included studies.

Study ID	Country	Study Design	Groups	Number of Each Group	Sample Size	Age	Male, n (%)	Any Other Heart Diseases	Hypertension	Coronary Artery Disease	Left Ventricular Dysfunction	MI
AbRahman et al., 2022 [39]	Malaysia	Self-controlled case-series study	BNT162b2	15,387,585	35,201,509 *	From 12 to 60 years and above	4,485,174	844 (13.83) ^d^	3061 (50.16) ^d^	NA	NA	NA
			CoronaVac	17,030,243			4,376,900					
			ChAdOx1	2,744,507			1,008,040					
Barda et al., 2021 [40]	Israel	Retrospective registry-based analysis	BNT162b2	884,828	1,769,656	39.333 (19.274) ^a^	461,590 (52)	31,836 (4)	94,819 (11)	NA	NA	NA
			Control	884,828		39.333 (19.274) ^a^	461,590 (52)	31,596 (4)	93,357 (11)			
Dagan et al., 2021 [26]	Israel	Subgroup analysis of Barda 2021 study	BNT162b2	884,828	1,769,656	39.333 (19.274) ^a^	461,590 (52)	31,836 (4)	94,819 (11)	NA	NA	NA
			Control	884,828		39.333 (19.274) ^a^	461,590 (52)	31,596 (4)	93,357 (11)			
Dickerman et al., 2022 [27]	US	Cohort Study	BNT162b2	216,836	433,672	67.667 (10.378) ^a^	200,908 (93)	59,153 (27) ^b^	137,265 (63)	NA	NA	NA
			mRNA-1273	216,836		67.667 (10.378) ^a^	200,908 (93)	59,266 (27) ^b^	140,774 (65)			
El-Shitany et al., 2022 [28]	Saudi Arabia	Cross-Sectional	BNT162b2	422	422	Any age groups ^c^	123 (27.8)	NA	94 (58.4)	NA	NA	NA
Kadali et al., 2021 [29]	US	Cross-Sectional	BNT162b2	803	803	From 18 to 90 years (Average 43 years)	108 (13.45)	NA	NA	NA	NA	NA
Kadali et al., 2021a (cross) [30]	US	Cross-Sectional	mRNA-1273	432	432	From 18 to 80 years (Average 43.76 years)	45 (10.42)	8 (1.85)	55 (12.73)	NA	NA	NA
Kaur et al., 2021 [31]	India	Cohort study	ChAdOx1	804	804	38.44 (11.47) ^e^	573 (71.27)	NA	73 (9)	5 (0.6)	NA	NA
Kaur et al., 2021b ^f^ [32]	Global	Retrospective registry-based analysis	BNT162b2	4201	4863	NA	NA	NA	NA	NA	NA	
			ChAdOx1	262								
			mRNA-1273	400								
Montano et al., 2022 [24]	Global	Retrospective registry-based analysis	ChAdOx1	34,643,783	452,016,608	From 18 to 65 years and above	266,008,304 (58.85)	NA	NA	NA	NA	NA
			Ad26.COV2.S	32,233,117								
			mRNA-1273	105,518,547								
			BNT162b2	279,620,827								
Patone et al., 2022 ^g^ [25]	England	Self-controlled case-series	ChAdOx1 (1st)	20,615,911	1st dose at least = 38,615,491	55.2 (14.8) ^e^	7191428 (34.9)	NA	NA	NA	NA	NA
			ChAdOx1 (2nd)	19,754,224		55.4 (14.7) ^e^	6900964 (34.9)					
			BNt162b2 (1st)	16,993,389		47.8 (21.7) ^e^	5401842 (31.8)					
			BNt162b2 (2nd)	11,972,733	Two doses = 32,095,748	55.5 (20.4) ^e^	3,906,666 (32.6)					
			mRNA-1273 (1st)	1,006,191		32.3 (9.4) ^e^	286,893 (28.5)					
			mRNA-1273 (2nd)	368,791		39.6 (7.3) ^e^	97,524 (26.4)					
Tan et al., 2021 ^g^ [33]	Singapore	Cohort	BNT162b2 (1st)	37,367	1st dose at least = 64,661	27.33 (11.119) ^a^	33,913 (90.8)	NA	NA	NA	NA	NA
			BNT162b2 (2nd)	37,162		27 (11.119) ^a^	33,766 (90.9)					
			mRNA-1273 (1st)	27,294	Two doses = 62,420	21 (2.9653) ^a^	25,661 (94.0)					
			mRNA-1273 (2nd)	25,258		21 (2.9653) ^a^	23,710 (93.9)					
Tobaiqy et al., 2021 [34]	Global	Retrospective registry-based analysis	mRNA-1273	336	3420	From 18 to 85 years and above	171 (50.9)	NA	NA	NA	NA	NA
			BNT162b2	1096			494 (45.1)					
			ChAdOx1	1988			952 (47.9)					
Truong et al., 2022 [35]	US and Canada	Retrospective study	BNT162b2	131	139	17.567 (18.728) ^e^	126 (90.6)	NA	NA	NA	NA	NA
			mRNA-1273	5								
			Ad26.COV2.S	1								
			Unknown	2								
Witberg et al., 2022 [36]	Israel	Retrospective registry-based analysis	BNT162b2	2,558,421	2,558,421	45.667 (24.463) ^e^	1,248,433 (49)	NA	7 (13) ^h^	1 (2) ^h^	1 (2) ^h^	NA
Wong et al., 2022 [37]	China	Retrospective study	BNT162b2 (1st)	1,308,820	1st dose at least = 2,264,679	45.7 (16.0) ^e^	584,158 (44.6)	657 (0.05) ^i^	177,913 (13.6)	253,883 (19.4)	NA	1662 (0.1)
			BNT162b2 (2nd)	1,116,677		45.9 (15.7) ^e^	502,740 (45)	513 (0.04) ^i^	148,978 (13.3)	210,140 (18.8)	NA	1347 (0.1)
			CoronaVac (1st)	955,859	Two doses = 1,938,237	55.3 (14.1) ^e^	439,928 (46)	881 (0.09) ^i^	225,107 (23.6)	343,413 (35.9)	NA	2238 (0.2)
			CoronaVac (2nd)	821,560		54.8 (13.9) ^e^	383,164 (46.6)	653 (0.07) ^i^	182,379 (22.2)	274,353 (33.4)	NA	1688 (0.2)
Dizbay et al., 2021 [38]	Turkey	Retrospective study	CoronaVac	1102	1102	37.667 (12.619) ^a^	352 (31.9)	13 (1.2)	48 (4.4)	NA	NA	NA

* Cumulative numbers include all types of vaccines administered to the population. Numbers may not add up to the total due to missing values. ^a^. Mean, and SD were calculated from median and IQR. ^b^. Cardiovascular disease included acute myocardial infarction, cardiomyopathy, cerebrovascular disease, coronary heart disease, heart failure, and peripheral vascular disease. ^c^. Not reported range or mean and SD. ^d^. In events groups. ^e^. Mean and SD. ^f^. Report data only in AE populations. ^g^. 1st = one dose at least; 2nd = two doses. ^h^. In the myocarditis group only. ^i^. Peripheral vascular disease.

**Table 2:** Meta-analysis outcomes.

Vaccine	Studies	Pooled Incidence	Lower CI	Upper CI	IR	Heterogeneity (I^2^)	*p*-Value
Pfizer	12	0.22%	0.07%	0.66%	22 per 10,000	99.96%	<0.001
Sinopharm	2	0.03%	0.00%	18.48%	3 per 10,000	99.96%	<0.001
Moderna	4	0.76%	0.04%	12.08%	76 per 10,000	99.92%	<0.001
CoronaVac	2	0.01%	0.00%	0.03%	1 per 10,000	99.59%	<0.001
AstraZeneca	3	0.04%	0.00%	0.98%	4 per 10,000	99.78%	<0.001

**Table 3:** Subgroup analysis.

Subgroup Analysis		Studies	Pooled Incidence	Lower CI	Upper CI	Heterogeneity (I^2^)	*p*-Value
Country	Asia	3	0.006%	0%	0.009%	92.8%	<0.001
	North America	3	11.95%	4.92%	18.98%	98.6%	<0.001
	Middle East	4	0.022%	0.001%	0.046%	97%	<0.001
	Europe	1	0.136%	0.134%	0.137%	-	-
Study Design	Cohort	2	0.692%	0%	2.04%	99.97%	<0.001
	Retrospective registry-based	5	0.023%	0%	0.053%	98.9%	<0.001
	Cross-sectional	2	5.25%	3.42%	7.07%	66%	0.086
	Case-series	2	0.071%	0%	0.198%	100%	<0.001
Sample Size	>2000	6	0.238%	0.183%	0.294%	99.99%	<0.001
	<2000	5	8.51%	3.58%	13.43%	98.05%	<0.001

## Data Availability

The data presented in this study are available in the article.
